# Supplementary material for: Body composition, physical fitness and physical activity in Mozambican children and adolescents living with HIV
Source: PLoS One. 2022 Oct 20;17(10):e0275963. doi: 10.1371/journal.pone.0275963 (PMC9584386; doi:10.1371/journal.pone.0275963)
Supplement: S1 Table — (DOCX) [file pone.0275963.s001.docx]

**S1 Table.** Descriptive data (mean±sd) of height, body mass index (BMI), sum of tricipital and subscapular skinfolds (∑skinfolds) and middle arm circumference (MAC).

| **Age**  **(years)** | **N** | **Height**  **(cm)** | **Weight**  **(kg)** | **BMI**  **(kg/m^-2^)** | **∑Skinfolds**  **(mm)** | **MAC**  **(mm)** |
| --- | --- | --- | --- | --- | --- | --- |
| ***Boys*** | | | | | | |
| 8 | 1 | 118.4 ± 5.1 | 21.1 ± 2.3 | 15.1 ± 0.9 | 12.1 ± 1.6 | 17.8 ± 1.5 |
| 9 | 5 | 130.6 ± 4.0 | 26.6 ± 1.3 | 15.6 ± 1.0 | 11.6 ± 2.8 | 16.8 ± 4.7 |
| 10 | 6 | 128.8 ± 6.3 | 28.7 ± 5.6 | 17.2 ± 2.8 | 14.1 ± 2.3 | 19.6 ± 1.8 |
| 11 | 4 | 137.8 ± 3.3 | 29.6 ± 1.5 | 15.6 ± 1.0 | 13.7 ± 4.1 | 20.7 ± 0.9 |
| 12 | 2 | 145.5 ± 4.9 | 37.3 ± 8.8 | 17.5 ± 2.9 | 15.4 ± 7.8 | 21.1 ± 2.9 |
| 13 | 7 | 144.1 ± 9.4 | 37.3 ± 7.7 | 17.8 ± 1.6 | 15.4 ± 2.5 | 22.2 ± 1.6 |
| 14 | 8 | 149.6 ± 6.4 | 38.4 ± 9.3 | 17.0 ± 3.3 | 13.4 ± 3.4 | 20.8 ± 2.2 |
| **Girls** | | | | | | |
| 8 | 7 | 120.7 ± 3.5 | 23.0 ± 4.2 | 15.8 ± 2.8 | 13.2 ± 2 | 18.4 ± 2.3 |
| 9 | 4 | 127.2 ± 5.3 | 26.1 ± 2.2 | 16.1 ± 0.6 | 14.2 ± 2.9 | 19.7 ± 1.6 |
| 10 | 4 | 137.5 ± 5.3 | 31.0 ± 5.6 | 16.3 ± 2.0 | 15.1 ± 7.7 | 20.2 ± 1.7 |
| 11 | 6 | 134.1 ± 5.4 | 27.9 ± 3.3 | 15.5 ± 1.9 | 12.9 ± 4.1 | 19.6 ± 2.2 |
| 12 | 4 | 134.3 ± 4.1 | 32.1 ± 5.3 | 15.8 ± 1.8 | 16.3 ± 3.4 | 20.6 ± 2.1 |
| 13 | 6 | 150.3 ± 8.6 | 42.3 ± 6.1 | 18.7 ± 1.4 | 21 ± 5,9 | 23.1 ± 1.5 |
| 14 | 5 | 149.8 ± 7.9 | 44.1 ± 8.3 | 19.5 ± 2.5 | 24 ± 11.5 | 23.4 ± 3.7 |
