# Supplementary material for: Body composition, physical fitness and physical activity in Mozambican children and adolescents living with HIV
Source: PLoS One. 2022 Oct 20;17(10):e0275963. doi: 10.1371/journal.pone.0275963 (PMC9584386; doi:10.1371/journal.pone.0275963)
Supplement: S2 Table — (DOCX) [file pone.0275963.s002.docx]

**S2 Table:** Descriptive data (mean±sd) of physical fitness tests of subjects with HIV

| **Age**  **(years)** | | **N** | | ***Sit and Reach***  ***(cm)*** | ***Sl jump***  ***(cm)*** | ***Curl up***  ***(reps/min)*** | ***Handgrip***  ***(kg)*** | |
| --- | --- | --- | --- | --- | --- | --- | --- | --- |
| ***Boys*** | | | | | | | | |
| 8 | 11 | | 26.8 ± 6.5 | | 111.9 ± 16.6 | 5.0 ± 4.3 | | 9.9 ± 3.6 |
| 9 | 5 | | 30.6 ± 3.2 | | 140.4 ±15.6 | 13.4 ± 3.4 | | 15.5 ± 2.2 |
| 10 | 6 | | 32.1 ± 6.2 | | 128.8 ± 12.2 | 9.2 ± 2.0 | | 16.9 ± 6.5 |
| 11 | 4 | | 31.3 ± 5.9 | | 136.0 ± 11.9 | 8.8 ±3.1 | | 17.0 ± 1.6 |
| 12 | 2 | | 18.0 ± 4,2 | | 133.5 ± 9.3 | 8.5 ± 0.1 | | 17.4 ± 3.7 |
| 13 | 7 | | 29.3 ± 4.6 | | 140.7 ± 19.5 | 12.1 ± 2.2 | | 20.4 ± 2.8 |
| 14 | 8 | | 30.0 ± 4.4 | | 141.1 ± 30 | 16.0 ± 8.9 | | 21.8 ± 4.2 |
| ***Girls*** | | | | | | | | |
| 8 | 7 | | 32.5 ± 4.1 | | 107.9 ± 14.8 | 7.3 ± 5.9 | | 10.3 ± 2.1 |
| 9 | 4 | | 33.1 ±2.2 | | 121.3 ± 8.8 | 8.0 ± 2.7 | | 12.9 ±4.1 |
| 10 | 4 | | 32.3 ± 8.1 | | 119..0 ± 13.1 | 8.5 ± 7.0 | | 16.2 ± 2.8 |
| 11 | 6 | | 31.7 ± 6.8 | | 114.5 ± 16.8 | 8.8 ± 4.7 | | 15.0 ± 3.5 |
| 12 | 4 | | 30.8 ± 6.9 | | 121.8 ± 15.4 | 7.3 ± 2.5 | | 17.5 ± 3.2 |
| 13 | 6 | | 34.5 ± 3.4 | | 148.8 ± 17.7 | 11.7 ± 1.4 | | 24.0 ±3.7 |
