# Supplementary material for: Body composition, physical fitness and physical activity in Mozambican children and adolescents living with HIV
Source: PLoS One. 2022 Oct 20;17(10):e0275963. doi: 10.1371/journal.pone.0275963 (PMC9584386; doi:10.1371/journal.pone.0275963)
Supplement: S3 Table — (DOCX) [file pone.0275963.s003.docx]

**S3 Table:** Descriptive data (mean±sd) of steps/day and average time spent in MVPA of subjects with HIV

| **Age** | **N** | **Steps /day** | **Time in MVPA/ day** |
| --- | --- | --- | --- |
| **Boys** | | | |
| 8 | 11 | 12 369 ± 2857.8 | 53.7 ± 14.1 |
| 9 | 5 | 11 586 ± 4163.4 | 52.2 ± 32.0 |
| 10 | 6 | 11 587 ± 4711 | 49.7 ± 20.4 |
| 11 | 4 | 12 828 ± 6885 | 51.8 ± 33.8 |
| 12 | 2 | 16 196 ± 1983 | 71.0 ± 7.1 |
| 13 | 7 | 11 160 ± 5239.8 | 44.0 ± 28.2 |
| 14 | 8 | 10 746 ± 4862.1 | 36.5 ± 22.0 |
| ***Girls*** | | | |
| 8 | 7 | 9 020 ± 2371.5 | 37.1 ± 13.7 |
| 9 | 4 | 11 622 ± 1103.2 | 45.0 ± 11.9 |
| 10 | 4 | 11 611 ± 3247.1 | 47.3 ± 12.1 |
| 11 | 6 | 8 273 ± 2700 | 26.5 ± 15.7 |
| 12 | 4 | 6 923 ± 3413.5 | 28.3 ± 20.3 |
| 13 | 6 | 11 024 ± 1184.7 | 44.5 ± 14.7 |
| 14 | 5 | 10 438 ± 1184.7 | 44.4 ± 8.7 |
