# Supplementary material for: Body composition, physical fitness and physical activity in Mozambican children and adolescents living with HIV
Source: PLoS One. 2022 Oct 20;17(10):e0275963. doi: 10.1371/journal.pone.0275963 (PMC9584386; doi:10.1371/journal.pone.0275963)
Supplement: S4 Table — (DOCX) [file pone.0275963.s004.docx]

**S4 Table:** Statistical procedures information

| **Figure / Table** | **Mean ± S. D** | | | | | | | | | | **Statistical Method used** | **P value** | **Sample** |
| --- | --- | --- | --- | --- | --- | --- | --- | --- | --- | --- | --- | --- | --- |
| **Figure: 1**  Individual position of each subject relative to reference standards |  | | | | | | | | | |  |  |  |
| a) height/age and gender |  |  |  |  |  |  |  |  |  |  | Percentile position of each subject in relation to WHO reference value |  | 43 Boys  36 Girl |
| b) BMI/age and gender |  |  |  |  |  |  |  |  |  |  | Percentile position of each subject in relation to WHO reference value |  | 43 Boys  36 Girl |
| c) MAC/age and gender |  |  |  |  |  |  |  |  |  |  | Percentile position of each subject in relation to Frisancho, 1990 reference value |  | 43 Boys  36 Girl |
| d) Sum of skinfolds/age and gender |  |  |  |  |  |  |  |  |  |  | Percentile position of each subject in relation to Frisancho, 1990 reference value |  | 43 Boys  36 Girl |
| **Table:1**  Comparison (mean±sd) of height, body mass index (BMI), sum of tricipital and subscapular skinfolds (∑skinfolds) and mid-arm circumference (MAC) between HIV+ and non-HIV+ Mozambicans boys and girls from urban and rural settings |  | | | | | | | | | | ANCOVA test with age as a covariate |  |  |
|  | **HIV+** | | | | **Urban** | | | | | **Rural** |  |  |  |
|  | **Boys** | | | | | | | | | |  |  |  |
| Height | 135.9 ± 1.2^a^ | | | | 142.7 ± 0.3^b^ | | | | | 140 ± 0.5^c^ |  | 0.000 | 43^d^ |
| BMI | 16.6 ± 0.4^ab^ | | | | 17.5 ± 0.1^a^ | | | | | 16.5 ± 0.2^b^ |  | 0.000 | 43^d^ |
| ∑Skinfolds | 13.5 ± 1.1^ab^ | | | | 14.1 ± 0.3^a^ | | | | | 12.3 ± 0.5^b^ |  | 0.005 | 43^d^ |
| MAC | 19.7 ± 0.3^ab^ | | | | 20.5 ± 0.9^a^ | | | | | 19.7 ± 0.1^b^ |  | 0.000 | 43^d^ |
|  | **Girls** | | | | | | | | | |  |  | |
| Height | 137.6 ± 1.3^a^ | | | | 143.8 ± 0.3^b^ | | | | | 141.7 ± 0.5^c^ |  | 0.000 | 36^d^ |
| BMI | 16.9 ± 0.5^a^ | | | | 18.6 ± 0.1^b^ | | | | | 17.3 ± 0.2^a^ |  | 0.000 | 36^d^ |
| ∑Skinfolds | 16.8 ± 1.9^ab^ | | | | 20.5 ± 0.4^a^ | | | | | 16.3 ± 0.7^b^ |  | 0.000 | 36^d^ |
| MAC | 20.6 ± 0.4^ab^ | | | | 21.4 ± 0.1^a^ | | | | | 20.8 ± 0.1^b^ |  | 0.000 | 36^d^ |
| **Table: 2**  Proportion (%) of stunting (low height for age), wasting (low weight for height) and overweight and obesity between HIV^+^ and non-HIV^+^ urban and rural Mozambican boys and girls |  | | | | | | | | | |  |  |  |
|  | **HIV+** | | | | **Urban** | | | | | **Rural** | Chi-square test value |  |  |
|  | **Boys** | | | | | | | | | |  |  |  |
| Low height for age | 34.9 | | | | 9.8 | | | | | 19.2 | 31.5 | 0.000 | 43^d^ |
| Low weight for height | 9.3 | | | | 8.9 | | | | | 9.6 | 23.9 | 0.001 | 43^d^ |
| Overweight + obesity | 4.6 | | | | 10.8 | | | | | 1.4 |  |  | 43^d^ |
|  | **Girls** | | | | | | | | | |  |  |  |
| Low height for age | 27.8 | | | | 8.0 | | | | | 12.1 | 17.5 | 0.000 | 36^d^ |
| Low weight for height | 11.1 | | | | 5.6 | | | | | 5.9 | 49.072 | 0.000 | 36^d^ |
| Overweight + obesity | 2.7 | | | | 18.1 | | | | | 3.7 |  |  | 36^d^ |
| **Table: 3**  Proportion (%) of subjects living with HIV by fitness category (fit and unfit). | **Fit** | | | | | **Unfit** | | | | |  |  |  |
|  | **Boys** | | | | | | | | | |  |  |  |
| Sit and reach | 83.7 | | | | | 16.3 | | | | |  |  | 43^d^ |
| Curl-up | 27.9 | | | | | 72.1 | | | | |  |  | 43^d^ |
| Hand grip | 18.6 | | | | | 81.4 | | | | |  |  | 43^d^ |
| Standing long jump | 25.6 | | | | | 74.4 | | | | |  |  | 43^d^ |
|  | **Girls** | | | | | | | | | |  |  |  |
| Sit and reach | 91.7 | | | | | 8.3 | | | | |  |  | 36^d^ |
| Curl-up | 19.4 | | | | | 80.6 | | | | |  |  | 36^d^ |
| Hand grip | 30.6 | | | | | 69.4 | | | | |  |  | 36^d^ |
| Standing long jump | 41.7 | | | | | 58.3 | | | | |  |  | 36^d^ |
| **Table: 4**  Comparison (mean±sd) of fitness tests for HIV^+^ subjects in urban and rural Mozambican boys and girls |  | | | | | | | | | | P values were generated from the ANCOVA test with age as a covariate. |  |  |
|  | **HIV+** | | | | **Urban** | | | | | **Rural** |  |  |  |
|  | **Boys** | | | | | | | | | |  |  |  |
| Sit and reach (cm) | 28.9 ± 0.9 | | | | 31.2 ± 0.3 | | | | | 31.2 ± 0.4 |  | 0.07 | 43^d^ |
| Curl-up (reps/min) | 11.3 ± 2.8^a^ | | | | 28.5 ± 0.8^b^ | | | | | 25.0 ± 1.1^c^ |  | 0.000 | 43^d^ |
| Hand grip (kg) | 17.3 ± 0.7^a^ | | | | 20.9 ± 0.2^b^ | | | | | 19.9 ± 0.3^c^ |  | 0.000 | 43^d^ |
| Standing long jump (cm) | 133.4 ± 3.1^a^ | | | | 146.8 ± 0.8^b^ | | | | | 128.0 ±1.3^a^ |  | 0.000 | 43^d^ |
|  | **Girls** | | | | | | | | | |  |  |  |
| Sit and reach (cm) | 33.2 ± 1.0 | | | | 34.1 ± 0.2 | | | | | 34.8 ± 0.2 |  | 0.2 | 36^d^ |
| Curl-up (reps/min) | 9.8± 3.1^a^ | | | | 26.6 ± 0.7^b^ | | | | | 21.1 ± 1.2^c^ |  | 0.000 | 36^d^ |
| Hand grip (kg) | 17.3 ± 0.7^a^ | | | | 20.3 ± 0.1^b^ | | | | | 19.5 ± 0.5^b^ |  | 0.000 | 36^d^ |
| Standing long jump (cm) | 126.1± 3.5^a^ | | | | 135.3 ± 0.8^b^ | | | | | 120.1 ± 1.3^a^ |  | 0.000 | 36^d^ |
| **Table: 5**  Comparison (mean±sd) of average number of steps per day and time spent in MVPA between HIV^+^ and HIV negative urban and rural Mozambican boys and girls |  | | | | | | | | | |  |  |  |
|  | **HIV+** | | | | **Urban** | | | | | **Rural** |  |  |  |
|  | **Boys** | | | | | | | | | |  |  |  |
| Step count (steps/day) | 11 962 ± 960^a^ | | | | 16 431 ± 359^b^ | | | | | 17 442 ± 1151^b^ | p values were generated from the ANCOVA test with age as a covariate | 0.000 | 43^d^ |
| MVPA (min/day) | 48.9 ± 5.4^a^ | | | | 70.6 ± 2.0^b^ | | | | | 68.8 ± 5.5^b^ |  | 0.001 | 43^d^ |
|  | **Girls** | | | | | | | | | |  |  |  |
| Step count (steps/day) | 9 805 ± 818^a^ | | | | 12 492 ± 241^b^ | | | | | 13 949 ± 818^b^ |  | 0.001 | 36^d^ |
| MVPA (min/day) | 38.7 ± 4.9 | | | | 51.1 ± 1.5 | | | | | 51.9 ± 4.4 |  | 0.05 | 36^d^ |
| **Table: 6**  Comparison (%) of subjects according to activity categories classified by the average number of steps per day |  | | | | | | | | | | p values resulted from the Chi-square test |  |  |
|  | **HIV+** | | | **Urban** | | | | | **Rural** | |  |  |  |
|  | **Boys** | | | | | | | | | |  |  |  |
| Insufficiently active | *46.5* | | | *24.0* | | | | | *20.9* | | *10.6* | *0.005* | 43^d^ |
| Active | 53.5 | | | 76.0 | | | | | 79.1 | |  |  | 43^d^ |
|  | **Girls** | | | | | | | | | |  |  |  |
| Insufficiently active | 77.8 | | 46.5 | | | | | 42.6 | | | 8.2 | 0.02 | 36^d^ |
| Active | 22.2 | | 53.5 | | | | | 57.4 | | |  |  | 36^d^ |
| Table 7: Comparison (%) of subjects according to activity categories classified by the average amount of time spent in MVPA |  | | | | | | | | | | p values resulted from the Chi-square test |  |  |
|  | **HIV+** | **Urban** | | | | | **Rural** | | | |  |  |  |
|  |  | **Boys** | | | | |  | | | |  |  |  |
| Insufficiently active | 67.4 | 40.7 | | | | | 39.5 | | | | 11.3 | 0.003 | 43^d^ |
| Active | 32.6 | 59.3 | | | | | 60.5 | | | |  |  | 43^d^ |
|  |  | **Girls** | | | | |  | | | |  |  |  |
| Insufficiently active | 91.7 | 69 | | | | | 70.2 | | | | 8.2 | 0.02 | 36^d^ |
| Active | 8.3 | 31 | | | | | 29.8 | | | |  |  | 36^d^ |

**Legend**: a) significantly different to b) and c); b) significant different to a) and b); c) significantly different to a) and c).

d) Sample referring to children and adolescents living with HIV
